# Lost opportunities in HIV prevention: programmes miss places where exposures are highest

**DOI:** 10.1186/1471-2458-8-31

**Published:** 2008-01-24

**Authors:** Ingvild F Sandøy, Seter Siziya, Knut Fylkesnes

**Affiliations:** 1 Centre for International Health, University of Bergen, Norway; 2 Department of Community Medicine, University of Zambia, Zambia

## Abstract

**Background:**

Efforts at HIV prevention that focus on high risk places might be more effective and less stigmatizing than those targeting high risk groups. The objective of the present study was to assess risk behaviour patterns, signs of current preventive interventions and apparent gaps in places where the risk of HIV transmission is high and in communities with high HIV prevalence.

**Methods:**

The PLACE method was used to collect data. Inhabitants of selected communities in Lusaka and Livingstone were interviewed about where people met new sexual partners. Signs of HIV preventive activities in these places were recorded. At selected venues, people were interviewed about their sexual behaviour. Peer educators and staff of NGOs were also interviewed.

**Results:**

The places identified were mostly bars, restaurants or sherbeens, and fewer than 20% reported any HIV preventive activity such as meetings, pamphlets or posters. In 43% of places in Livingstone and 26% in Lusaka, condoms were never available. There were few active peer educators. Among the 432 persons in Lusaka and 676 in Livingstone who were invited for interview about sexual behaviour, consistent condom use was relatively high in Lusaka (77%) but low in Livingstone (44% of men and 34% of women). Having no condom available was the most common reason for not using one. Condom use in Livingstone was higher among individuals socializing in places where condoms always were available.

**Conclusion:**

In the places studied we found a high prevalence of behaviours with a high potential for HIV transmission but few signs of HIV preventive interventions. Covering the gaps in prevention in these high exposure places should be given the highest priority.

## Background

Many efforts at HIV prevention in sub-Saharan Africa have been aimed at high risk groups as these are believed to contribute disproportionately to the spread of the epidemic [1-3]. In countries with a relatively low HIV prevalence in the general population, much of the HIV transmission usually occurs from high risk/core groups (e.g. sex workers) to bridging populations (e.g. male clients) to the general population (e.g. low risk female partners). In settings with high prevalence in the general population, more transmission takes place among non-core individuals, but high risk groups can still be an important source of new infections [4-6]. In a study from rural Zimbabwe, 20% of infections among men in the general population were estimated to be due to sexual contact with sex workers [1]. Several examples show that interventions such as syndromic management, presumptive or prophylactic treatment of STIs, dedicated STI clinics, peer education and condom distribution targeted at sex workers have been successful in achieving increased condom use [7-16] and reduced incidence of STIs [7-9,12,13,17,18] and HIV [8,18-20] in this high risk group, and some have also found reduced prevalence of STIs [17] and/or HIV [8,18] among their clients. Very few of these trials have, however, had randomized designs and comparable control groups. There is evidence that such interventions had an effect on the HIV incidence in the general population of Thailand [8,18] and Cambodia [21,22], but the potential impact on the population incidence in countries with much higher HIV prevalences, as in sub-Saharan Africa, is more uncertain [23]. A problem with interventions targeting specific groups is that they may cause stigmatisation of already-marginalized individuals. Furthermore, in many countries in Africa transactional sex and frequent partner changes are quite common among women who do not identify themselves as sex workers [1,24-26]. Using mathematical models, Boily et al. found that for epidemics with a high basic reproductive number, interventions targeting core groups should be supplemented by general population approaches early in the epidemic [5].

Weir et al. have proposed that prevention should be focused on places where exposure to HIV can be assumed to be very high and have described a method for identifying such places rapidly (called "Priorities for Local AIDS Control Efforts", abbreviated PLACE-method) [23]. The assumption is that in countries where the HIV prevalence is high, focusing on high risk places such as venues where people meet new sexual partners would be even more effective in reducing the HIV transmission rate than targeting interventions just at perceived high risk groups. It may also cause less stigmatisation [27]. Accordingly, studies of such high risk sites to assess the coverage and intensity of interventions are of particular importance. Studies of this type from sub-Saharan Africa have found few HIV interventions or condoms in such venues [23,28-30]. However, PLACE-studies conducted in East London, South Africa, in 2000 and 2003 showed an increase in condom use and a reduction in numbers of recent partners during a 3 year period, and this could possibly be related to a community-based intervention targeting high risk venues [31].

Despite numerous condom promotion campaigns in sub-Saharan Africa, condom use with non-regular partners for prevention of STIs, including HIV, is still relatively low (< 50%) [32-35]. An increase in condom use for casual sex has been observed in Zambia and Zimbabwe [34,36-38], together with other changes in sexual behaviour. This is probably related to the observed declines in HIV prevalence. However, the prevalence among young people (a proxy of incidence) in both these countries is still high. It seems that condom use must reach a certain level to have a major influence on the epidemic [6,8,33]. Several studies indicate that making condoms easily available in places where people meet sexual partners may be critical for increasing the probability that people will use them [28,39-42]. However, despite many condom promotion campaigns, condoms are seldom found in bars, discos or guest houses where people meet potential sexual partners [23,28-30].

A growing number of studies from southern Africa have revealed signs of decline in the HIV epidemic [37,38,43-48], but have also shown marked local differentials within countries [45,47,49]. An example from Zambia is the difference between trends appearing in two major cities, Livingstone and Lusaka. In Livingstone, HIV surveillance among antenatal clinic (ANC) attendees conducted since 1993 has shown a high and stable HIV prevalence of around 30% among women below the age of 25 years. In contrast, the HIV prevalence in Lusaka was very high in the early 1990s, but has declined during the past decade according to both ANC-based HIV surveillance and population-based surveys from Chelston township, i.e. from 22.5% in 1995 to 12.5% in 2003 in women in the general population aged 15–24 years [50,51]. Unfortunately, no system for monitoring the coverage and intensity of preventive interventions has been put in place until recently in either city, so there is insufficient information to explore the relationship between differentials in transmission trends and past preventive efforts. Although HIV prevention programs appear to have been successful in Chelston, it is vital to sustain the energy in both cities. We investigated signs of current preventive efforts and apparent gaps in places where people meet new partners in these two urban settings approximately 20 years after the first prevention programs were launched.

## Methods

We wanted to study high risk places in Lusaka, the capital city of Zambia, and in Livingstone, a border city and the main tourist spot in Zambia, which are two urban communities where the HIV epidemic has developed differently. We selected townships in the two cities for which we have data about HIV prevalence over time. The ANC-based HIV surveillance in Livingstone includes data from the health clinics in Maramba, Dambwa and New Boma, and the results from these three clinics are pooled in the surveillance reports. In Lusaka, we chose Chelston as our study community as we have both population-based and ANC-based prevalence data from this township. According to the 2000 population census Chelston had a population of 33,700 and the three townships in Livingstone had a total population of 41,800. Both cities have high unemployment rates, but we observed worse housing and sanitary conditions, indicating higher poverty levels, in the three study communities in Livingstone than in Chelston. Livingstone had many sherbeens (informal drinking places operating illegally without a license to serve alcohol), but none were found in Chelston.

The PLACE-method was used to collect the information. During the first phase of the study people who knew the selected communities well because they lived or worked there, such as young people encountered in the streets, health personnel, taxi drivers, shop staff and bar workers, were interviewed about places where local people meet new sexual partners. The informants were people that the interviewers met as they walked systematically through the study areas. The target number of informants during the first phase was 200 in Chelston and 400 in Livingstone as a bigger area would be covered in Livingstone. The study team also looked for places where people gathered and it seemed likely that new partnerships were formed.

During the second phase, the interviewers tried to find all the sites that had been mentioned. As informants in Livingstone, with just a few exceptions, only mentioned places in Maramba, Dambwa and the city centre, we decided to examine only venues in these areas. In Chelston, most of the places mentioned were within Chelston, and we only visited these. In those places that were found, one person who knew the site well (a bar worker or a regular guest) was interviewed about what activities occurred there and the availability of condoms and educational materials.

The last part of the study consisted of interviews on sexual behaviour and partnership establishment with a sample of individuals who socialized at selected venues. All sites mentioned by more than 10 informants during the first phase, and a random sample of 30% of the remaining sites were selected for interviews (with the probability of selection proportional to the estimated number of guests on an average night). The interviewers approached individuals who were standing along two imaginary diagonal lines connecting the four corners of the room, and tried to interview as many as possible in each venue visited.

We excluded churches and schools that had been identified in the first phase from the second and third phases of the study because the authorities were reluctant to distribute condoms, and we did not expect them openly to admit that people met sexual partners in schools or churches.

In addition, the interviewers recorded observations and concerns that were repeatedly mentioned in the venues. We also interviewed one nurse, one counsellor and 1–2 peer educators at three of the health clinics in the townships, and staff of the NGOs who ran HIV preventive activities in the study areas. All the interviews were performed between September and December 2005.

### Statistical analysis

Statistical analyses were performed using SPSS version 13-0. The results of the interviews with people socializing in the venues were weighted by multiplying by the estimated number of guests at busy hours and dividing by the number of interviews conducted. We performed a chi-square test of independence to test for a relationship between number of partners in the last month and engaging in transactional sex among women. A p-value less than 0.05 indicated a significant association between the number of sexual partners and engaging in transactional sex.

### Ethical aspects

We only interviewed adults 18 years or older. All interviews were anonymous and based on oral consent, and the informants were assured that the information they provided would not be linked to them or to the specific site. The protocol was approved by the Research Ethics Committee of the University of Zambia (Ref no. 012-08-05).

## Results

During the first phase of the study, 275 informants in Lusaka and 434 in Livingstone were asked where people met new sexual partners. Seventy-eight places in Lusaka and 147 situated in Maramba, Dambwa and the city centre of Livingstone were identified. In Lusaka, most of the places were bars or restaurants. The rest were schools, night clubs, guest houses, churches, a cinema hall and a bus stop. In Livingstone, 50% of the places were sherbeens, 37% bars and restaurants, and the rest were guest houses and night clubs (Table 1). Approximately 40% of the venues were reported to have between 10–25 guests during busy hours, and another 40% of the venues had 26–50 guests.

**Table 1:** Venues mentioned by informants during the first phase

		**Lusaka**		**Livingstone**	
		%	N (sites)	%	N (sites)

Type of place	Sherbeen	0	78	50	147
	Bar/restaurant	76		37	
	Night club	4		5	
	Guest house	3		7	
	School	5		0	
	Bus station	1		0	
	Church	10		0	
	Cinema hall	1		0	
					
Place verification	Venue found	82	78	78	147
	Venue found, but no venue representatives willing to be interviewed	3		1	
	Venue not found	0		12	
	Venue closed temporarily	3		0	
	Venue closed permanently	3		1	
	Venue not visited	10		10	

### HIV preventive activities in venues

HIV preventive activities such as meetings and distribution of pamphlets had taken place in fewer than 5% of the venues, and HIV-related posters had been present in 17% of the venues in Livingstone and 3% in Lusaka (Table 2). Only a third of the venues were reported always to have condoms available. When asked to produce condoms, the staff in 42% and 26% of the venues in Livingstone and Lusaka respectively could do so (Table 3). Condoms were never available in 26% of the venues in Lusaka and 43% in Livingstone (Table 2). However, respondents in 84% of the venues in Lusaka and 63% in Livingstone stated that it was possible to find condoms within 10 minutes of the site.

**Table 2:** Responses to interview with venue representatives (one per site)

			**Lusaka**		**Livingstone**	
			%	N (sites)	%	N (sites)

Staff or patron agreed to be interviewed			97	66	99	116
Men meet new female sexual partners here			72	64	82	115
Women meet new male sexual partners here			73	64	82	114
Men meet new male sexual partners here			11	64	0	114
Sex workers operate here			73	64	80	115
						
Among men who come to this venue	--- find new sexual partners here	None	28	61	21	114
		Some	35		73	
		Most	37		6	
	--- appear to be paying for sex	None	31	61	21	114
		Some	34		73	
		Most	35		7	
Among women who come to this venue	--- find new sexual partners here	None	33	61	19	113
		Some	30		70	
		Most	37		11	
	--- appear to be selling sex	None	32	62	19	113
		Some	28		69	
		Most	40		11	
HIV preventive activities that have taken place in venue^1^		Lectures/seminars	2	58	4	115
		Pamphlets/leaflets	3	58	3	115
		Posters	3	58	17	115
How often condoms available to guests in venue^1^	Always		28	58	31	114
	Sometimes		47		26	
	Never		26		43	
Condoms reported to be available in venue at time of interview^1^			29	58	44	115

N varies because of missing responses to some questions.

^1 ^Including only sherbeens, bars/restaurants, night clubs, guest houses

**Table 3:** Percentage of venues where interviewers observed condoms

		**Lusaka**		**Livingstone**	
		%	N (sites)	%	N (sites)

Condoms observed by interviewer		26	58	42	115
					
Percentage of venues^1 ^where condoms observed among ...	Venues where condoms said always to be available	75	16	94	35
	Venues where condoms said sometimes to be available	11	27	47	30
	Venues where condoms said never to be available	0	15	2	49

^1 ^Including only sherbeens, bars/restaurants, night clubs, guest houses

### Individuals socializing

During the third phase of the study 432 persons in Lusaka and 676 in Livingstone were invited for interview. The refusal rates were 2.8% in Lusaka and 2.5% in Livingstone. Eighty-five percent of the respondents in Lusaka and 77% in Livingstone were males. Almost all (> 99%) were from the city where they were interviewed. The average ages were 21.1 (SD 3.3) and 24.9 (SD 5.6) years for females (p < 0.001) and 26.9 (SD 5.1) and 29.9 (SD 6.6) years for males (p < 0.001) in Lusaka and Livingstone, respectively, and the average numbers of years of school attendance were 11.6 (SD 1.7) and 9.9 (SD 2.2) for women (p < 0.001) and 11.5 (SD 2.5) and 11.4 (SD 2.4) for men.

### High level of sexual risk behaviour

Half the men in both cities stated that they had come to the venues to meet a sexual partner. Among women, 66% in Livingstone and 8% in Lusaka reported such intentions, and 85% and 60% respectively reported ever having met a sexual partner in the venue where they were interviewed (Table 4). In 99% of the venues at least one person reported having previously met sexual partners in the place where he/she was interviewed. In Livingstone, 93% of women said this had happened during the previous week, whereas in Lusaka, 51% reported the same. Half the women interviewed in Lusaka and 87% in Livingstone had received money in exchange for sex during the 3 months prior to the survey, but the interviewers observed that more women were approaching men and seemed to be negotiating sex than admitted it. The median number of partners in the last month preceding the survey was 3 (IQR 2–4) for men in both cities, 3 (IQR 2–4) for women in Livingstone and 2 (IQR 1–3) for women in Lusaka. A high proportion of these partners were new for women in both Livingstone (77%) and Lusaka (50%). We also found that women who had had 3 or more sexual partners in the previous month were more likely to engage in transactional sex (p < 0.001).

**Table 4:** Responses from individuals socializing at places where people meet new sex partners

		**Males**				**Females**			
		**Lusaka**		**Livingstone**		**Lusaka**		**Livingstone**	

		**%**	N*	**%**	N*	**%**	N*	**%**	N*

Came to meet a sexual partner		53	229	51	413	8	61	66	138
Ever met a sexual partner here		68	358	63	508	60	62	85	149
									
Used a condom the last time had sex with a partner from this venue		85	234	64	315	81	37	52	126
									
Any new sexual partner last 4 weeks		76	354	78	500	70	62	87	149
Used a condom the last time had sex with one of these new partners		86	265	61	386	88	43	55	129
									
How often used condom with new partners during the previous 4 weeks	Always	77	258	44	378	77	43	34	125
	Sometimes	22		44		23		58	
	Never	1		13		0		8	
									
Have brought a condom		45	356	36	503	5	61	46	151
Condom shown if claimed to have brought		93	150	94	179	25	4	83	69
Condom shown (out of all)		39	366	33	517	2	65	37	154
									
Self-perceived risk of HIV	None	35	350	33	505	23	61	37	151
	Moderate	45		36		34		36	
	High	12		20		25		15	
	Very high	7		10		18		12	
									
Discussed with anyone in the last 6 months how to prevent becoming HIV infected		86	273	83	498	85	54	65	150
									
If yes, with whom?	Parents	8	235	2	413	2	46	3	97
	Grandparents	4		2		13		4	
	Brothers/sisters/cousin	38		15		15		20	
	Spouse	39		42		50		27	
	Friends	94		80		94		92	
	Peer educators	11		19		9		16	
	Health personnel	18		29		24		22	
									
Have received money in exchange for sex in the past 3 months (women)		-		-		50	62	87	150
Used a condom last time received money in exchange for sex (women)		-		-		85	27	54	127
									
Given money or gifts in exchange for sex in the past 3 months (men)		74	356	81	499	-		-	
Used a condom last time paid for sex (men)		92	264	69	405	-		-	
Had sex with man during the past 4 weeks (men)		0	348	0	494	-		-	

N varies because of missing responses to some questions.

*Individuals socializing at venues

### Condom use

More than 80% of men and women in Lusaka claimed to have used a condom both during the last sexual intercourse with a partner from the venue where they were interviewed and with the last new partner, whereas only half the women and approximately 60% of the men in Livingstone reported the same. The proportion of respondents who had used a condom with all new partners in the previous month was also much higher in Lusaka, and the same applied to condom use with clients for women who admitted receiving gifts for sex (Table 4). About three quarters of men and 67% of women with more than 9 years of schooling used a condom with the last new partner compared to 43% and 20% respectively with 0–7 years of education (p < 0.001). The proportions who reported not using a condom with the last new partner were 14% and 39% among men and 12% and 45% among women in Lusaka and Livingstone respectively. Not having a condom at hand, trusting the partner, dislike of condoms, and conviction that condoms can break, were the most common reasons mentioned. The majority (68%) of male respondents in Lusaka believed that condoms were very effective in preventing HIV, but the others were less convinced. Twenty-nine percent of men and 34% of women in Livingstone and 17% of men and 44% of women in Lusaka expressed low trust in the effectiveness of condoms.

**Figure 1:**
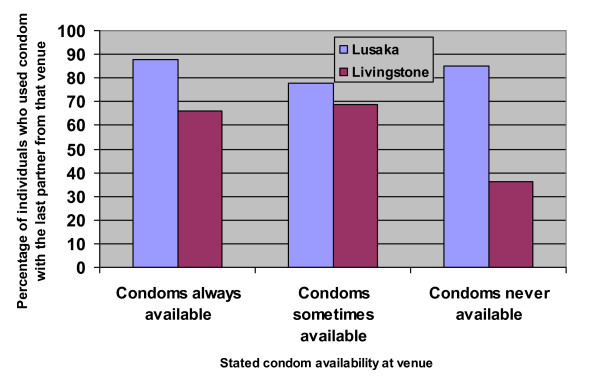
**Condom use with the previous partner in relation to condom availability at the venue**. Proportion reporting condom use with the previous sexual partner from a specific venue in relation to how often condoms were reported to be available at the same venue, men and women combined.

The majority of women said that their partners had brought the last condom they used. More women in Livingstone than in Lusaka had a condom in their pocket when asked to show the interviewers one, whereas around a third of men in both cities could prove that they had brought a condom (Table 4). Women in Livingstone who had a condom with them were more likely to have used a condom with the last new partner than those who did not have a condom (OR 2.72; 95% CI 1.27–5.84). Condom use in Livingstone was also higher among individuals who socialized in places where condoms were declared always to be available and in places where condoms were observed by the interviewers (p = 0.001), and this was especially the case for men who were not convinced that condoms were effective in preventing HIV transmission. In Lusaka, reported condom availability was not associated with condom use (Figure 1).

### Current HIV preventive programs in the townships

In both Lusaka and Livingstone, the local health clinics ran VCT and youth peer education programs. In addition, some NGOs ran such programs and distributed condoms. In both cities, a number of youth peer educators had been trained during the previous 2–3 years, but only a handful were still active in the townships we studied. For example, 2 years after 20 peer educators were trained by one NGO in Lusaka, only one was still involved. Most of the peer educators had quit because of lack of incentives, follow-up, material or mental support, or fringe benefits such as reduction in fees at the health clinic from which they were working. Many of the peer educators were also frustrated that they were not involved in planning prevention and health care services for young people. Those peer educators who were still active worked primarily from their offices and rarely did outreach activities. None of the peer programs currently targeted places where people met sexual partners, and no other programs did this at the time of the survey. The posters and leaflets that were found in a few of the venues had been distributed by peer educators some time previously.

None of the clinics in the townships in our study currently had programs targeting sex workers, and only one NGO initiative, Corridors of Hope, targeted this group in Livingstone in 2005. They reported that they were offering approximately 2000 sex workers in Livingstone free STI-services and VCT, promoting condom use, and training sex workers as peer educators (Program Director, Corridors of Hope (CoH), Family Health International). According to a survey conducted by CoH among female sex workers in 2003, 51% in Livingstone were registered with the program [52]. In 2006, however, they closed down all their activities in the city.

## Discussion

This study successfully identified venues at which there was a high risk of HIV exposure among individuals socializing. Most of the guests interviewed reported having met a sexual partner in the venue where they were interviewed, and the median number of sexual partners reported during the month prior to the study was high. Our study revealed missed opportunities in prevention, as very few of these venues seemed ever to have been exposed to HIV preventive activities, and we found no current interventions that specifically targeted these high-risk venues. In comparison, similar PLACE-studies conducted in two other urban areas in Zambia, i.e. Mongu and Kapiri Mposhi, in 2005 found that one third and half the high-risk venues reported previous HIV prevention activities, respectively [53,54]. The current study also revealed major gaps related to recruitment, continuous training and follow-up of youth peer educators.

The respondents reported a higher median number of sexual partners in the previous month than men and women in the general population in Chelston, who reported a median of 1 for the previous year in 2003 [unpublished data]. The number was also higher than the median of 1 found among men and women in the PLACE-studies in Mongu and Kapiri Mposhi [53,54]. Reported condom use among respondents in both Lusaka and Livingstone was higher than has been reported with casual partners in the Sexual Behaviour Survey 2005 (50% in urban areas) [34] and in the PLACE-studies from Mongu and Kapiri Mposhi (< 25%) [53,54], and in Lusaka the level was slightly higher than in the population-based survey from Chelston (70%) [Unpublished data]. This may reflect the higher partner turnover in the current study.

The lack of association between condom availability and condom use in Lusaka may be partly because condoms were allegedly nearly always available nearby, and higher trust in the protective effect of condoms among men in Lusaka made them willing to walk further to obtain condoms when they needed them. Most women in both cities relied on their partner to have a condom, and considering that a higher percentage of women in Livingstone than in Lusaka had a condom in their pocket (37% vs. 2%), their attitude to condoms did not seem to be decisive. In both cities not having a condom at hand was the most usual reason given for not using one with the last new partner or the last paying partner. After decades of condom promotion campaigns, it is thought-provoking that so many of the respondents expressed low trust in the effectiveness of condoms. Influential religious and political leaders who have emphasized the theoretical risk of HIV transmission when using a condom may have contributed to this [55-57]. It is encouraging that making condoms readily available seemed partially to compensate for sceptical attitudes among men in Livingstone. Since many of those who are out socializing also consume alcohol, making them less cautious, and as sexual intercourse is often not planned, it is important to make it easy for people to protect themselves. Providing condoms at venues where people socialize is therefore a simple but potentially effective structural intervention for preventing HIV transmission. Condoms can easily be placed in toilets and in rooms of guest houses and hotels so that people can obtain them out of public sight.

Transactional sex was very common among respondents in both cities, especially in Livingstone, which might reflect the higher level of poverty there. Numerous studies from sub-Saharan Africa have shown that many women who engage in transactional sex do not identify themselves as commercial sex workers, nor are they seen as such by the rest of the community. Receiving money and gifts for sex may be among the few available ways for women to secure themselves and their families financially when other sources of income are not sufficient [24,25,58-62], but the women may also have some hope that such relationships will evolve to become more lasting, i.e. marriage. The desperate need for money and the interest in a long-term relationship often put them in a weak position for negotiating condom use [24-26,59-61].

There have generally been very few preventive interventions in Zambia targeting sex workers with dedicated clinics and peer education, and Corridors of Hope, the only provider in Livingstone, has now closed down its activities. There is a need for political will to launch such interventions which have been shown to be effective in reducing HIV incidence among sex workers. A limitation of such programs is, however, that they may not reach women who only periodically engage in transactional sex, although they also may have a high partner turnover and many concurrent partners and may seldom use condoms [59]. Interventions targeting high risk places in addition to high risk groups are likely to reach a higher proportion of these women. This means that all venues where people meet sexual partners should be targeted, since women who perceive themselves as professional sex workers may operate in other establishments and other neighbourhoods than women who engage in transactional sex more infrequently [59]. There is currently a lack of strong evidence for the hypothesis that providing condoms and health educational materials in venues where people meet new sexual partners has an impact on HIV incidence in the general population. However, although no randomized field trials have been conducted that directly compare interventions targeting places with those targeting specific groups, it makes sense that approaches that also include women who engage in high risk behaviours only periodically, and their male partners, will be more effective against mature generalized epidemics.

Half the venues identified in Livingstone were sherbeens. In contrast, such places were non-existent in Chelston. This difference may reflect a different level of law enforcement in respect of alcohol regulations, or simply a different drinking culture. We do not have detailed data about the socioeconomic conditions in the townships we studied, but the available information from the Central Statistical Office is consistent with the observation of a high level of poverty in both cities.

We are convinced that we have a representative sample of venues where people meet sexual partners, as we tried to visit all the places mentioned and were able to find representatives willing to be interviewed in almost all of them. During the third phase, however, a convenient sampling method is used in all PLACE-studies. People who were reluctant to be interviewed could withdraw from the space near the interviewers. This means that although we recorded a very low refusal rate and tried to follow a predetermined sampling technique, we may not have interviewed a representative sample of the guests. The fact that some of the questions had a higher proportion of missing values than others, e.g. "Why did you come here today? – to meet a sexual partner?", could indicate partial refusals, but these questions did not appear to be more intimate than questions with a higher response rate. In any case, it is impossible to judge whether the successfully interviewed were engaging in greater or lesser risk behaviours than guests who were not interviewed. As in any study on sexual behaviour, it is also possible that the respondents were both under- and over-reporting certain behaviours. We found signs that some women who denied intentions to meet a sexual partner were not speaking the truth. Condom use may have been over-reported. However, the information that could be validated about condoms turned out to be reliable; in most of the places where the staff/patrons claimed that condoms were always available, and among most of the men who said they had brought a condom, this was verified.

The PLACE-method is intended for rapid assessment of the current situation and for making recommendations for the future based on such assessments, and not for drawing causal inferences. We only have data from one point in time, and it is therefore impossible to say whether any of the differences we found in the data in Lusaka and Livingstone could explain why the epidemics have had different courses in the two cities. Although few signs of HIV preventive programs were revealed at the time of the study, we cannot exclude the possibility that both cities may previously have been exposed to intensive campaigns against HIV in such venues.

## Conclusion

Many missed HIV preventive opportunities were identified in places where exposure is highest. Covering this gap would probably have a high impact on a short term basis, and the need to establish a monitoring and evaluation system in this regard is urgent. The present findings are to be followed up in the areas studied through an intervention comprising condom distribution and peer education among young people. To curtail transmission further, however, we believe that interventions with more long-term perspectives are badly needed, for example offering social and economic support programs that target women being forced into sex work.

## List of abbreviations used

ANC Antenatal clinic

HIV Human immunodeficiency virus

NGO Non-governmental organization

PLACE Priorities for Local AIDS Control Efforts

STI Sexually transmitted infection

## Conflicts of interest

The author(s) declare that they have no competing interests.

## Authors' contributions

IFS prepared the proposal, coordinated and supervised the survey, and conducted interviews with health personnel and peer educators, analysed the data, interpreted the findings and wrote the manuscript. SS helped develop the protocol, assisted in the supervision of data collection, and took active part in revising the manuscript. KF made substantial contributions to the conception of the survey and took active part in interpreting the results and revising the manuscript. All authors read and approved the final manuscript.

## Pre-publication history

The pre-publication history for this paper can be accessed here:


